# Climate emergency coping scale: development and validation of a multidimensional scale

**DOI:** 10.3389/fpsyg.2025.1665867

**Published:** 2025-11-20

**Authors:** Cintia Díaz-Silveira, Felisa Latorre, Paula Ganitsky-Chemby, Francisco Burgos-Julián, Maria Luisa Vecina

**Affiliations:** 1 Universidad Rey Juan Carlos Facultad de Ciencias de la Salud, Alcorcón, Spain; 2 Universidade de Santiago de Compostela, Santiago de Compostela, Spain; 3 Universidad Complutense de Madrid Facultad de Psicologia, Pozuelo de Alarcón, Spain

**Keywords:** coping, climate emergency, functional, dysfunctional, individual, social

## Abstract

Climate emergency poses not only environmental and economic challenges but also serious psychological consequences, contributing to growing levels of distress, anxiety, and helplessness. Despite increasing recognition of these effects, there is a lack of validated tools to assess how people cope emotionally and behaviorally with the climate crisis, especially in distinguishing between individual and social strategies. To address this gap, we developed and validated the Climate Emergency Coping Scale (CECS) through four studies conducted with Spanish samples. Study 1 (*n* = 520) used qualitative analysis to identify coping strategies from open-ended responses, forming the basis for item generation. Study 2 (*n* = 242) piloted the preliminary version to test its factorial structure and refine items. Study 3 (*n* = 1,021) explored the factorial structure of the final 12-item scale using exploratory factor analysis, and Study 4 (*n* = 1,064) confirmed the three-factor model—functional-individual, functional-social, and dysfunctional coping—while providing evidence of reliability, convergent, discriminant, incremental validity, and measurement invariance across gender, age, and education. The CECS offers a psychometrically robust instrument for assessing how individuals and communities cope with the emotional impact of the climate emergency. This scale provides a valuable framework for future research and intervention aimed at promoting adaptive coping and collective efficacy in the face of global environmental challenges.

## 1 Introduction

The climate emergency constitutes not solely an environmental and economic dilemma but also a profound psychological and emotional obstacle for numerous individuals (IPCC, 2022). The term *climate emergency* refers to the recognition that climate change has reached a critical point requiring urgent and unprecedented action to avoid irreversible ecological and societal damage (Ripple et al., 2020; IPCC, 2022). Unlike the broader concept of climate change, which primarily describes environmental alterations, the notion of emergency emphasizes its immediacy, systemic nature, and moral implications. It encompasses the escalating frequency of extreme weather events, biodiversity loss, and disruptions to human livelihoods, creating a chronic and collective stressor. These global and enduring treats shape individuals’ emotional experiences—evoking anxiety, guilt, grief, or anger—and call for coping responses that integrate both personal adaptation and collective engagement. Therefore, understanding coping within the context of the climate emergency requires addressing not only how individuals regulate emotions but also how they act, connect, and find meaning amid a perceived global crisis.

The ramifications of the climate emergency on mental health manifest in various ways, encompassing both direct and indirect effects (Daeninck et al., 2023). Direct consequences of the climate emergency pertain to individuals’ firsthand exposure to geophysical alterations with the risk of mental health disorders, including post-traumatic stress disorder, sleep disturbances, depressive states, and even suicidal thoughts (Cianconi et al., 2020; Charlson et al., 2021; Hayes et al., 2018). Furthermore, the indirect consequences of the climate emergency encompass psychological and emotional responses that do not constitute mental disorders, but rather include uncertainties about the future and its anticipated effects, the observation of losses experienced by others, and the witnessing of adversities occurring in various parts of the world (Ojala et al., 2021). These may include distress, worry, anxiety, anger, fear, grief, frustration, stress, denial, hopelessness, helplessness, guilt, cynicism, fatalism, and a paralyzing inability to act (Clayton, 2017; Coffey et al., 2021; Contreras et al., 2024; Lawrance et al., 2022; Marczak et al., 2023; Martiskainen et al., 2020; Ojala et al., 2021; Watts et al., 2021; Zaremba et al., 2023). Psychological associations have collectively acknowledged these climate-related emotional responses as legitimate reactions to the environmental emergency, while emphasizing the need to provide appropriate support to ensure that individuals and communities can cope with these feelings healthily and constructively (Lawrance et al., 2022; Verplanken et al., 2020).

The *Transactional Model of Coping* posits that individuals employ a range of strategies in response to stressors, based on how they appraise a given situation (Lazarus and Folkman, 1984). Understanding how people psychologically cope with the evolving climate crisis is crucial, as “humanity’s ability to adapt physically will depend in part on how well people adapt psychologically” (Hamilton and Kasser, 2009, p. 10). A growing body of research shows that different coping strategies influence mental health, pro-environmental behavior, resilience, and climate action (Helm et al., 2018; Mah et al., 2020).

Coping frameworks were first applied to the climate context by Homburg and Stolberg (2006), and later expanded by others (Ojala, 2012a, 2012b, 2013; Van Zomeren et al., 2010). Problem-focused coping involves directly addressing the stressor, for example, through activism, lifestyle changes, or seeking climate-related information (Lazarus and Folkman, 1984; Ojala, 2013). Emotion-focused coping refers to strategies aimed at alleviating emotional discomfort without addressing the root cause, including avoidance, disengagement, displacing responsibility, venting, or denial (Lazarus and Folkman, 1984; Ojala, 2013; Stanisławski, 2019). Later, Ojala (2012b) introduced meaning-focused coping in the context of climate change. This form of coping promotes positive psychological states (Folkman, 1997, 2007) and adaptive cognitions that enhance emotional resilience—such as spirituality, the belief that individual or collective actions matter, and the expression of existential hope (Daeninck et al., 2023; Ojala, 2012a).

Building on these theoretical foundations, coping with climate emergency could be conceptualized not only in terms of the strategy’s function (i.e., whether it is adaptive or maladaptive), but also in terms of its level of action. Global, chronic, and socially shared stressors, such as the climate crisis, often elicit not only individual self-regulatory responses but also socially oriented coping strategies involving collaboration, advocacy, and mutual support. This perspective aligns with research on collective coping (Lyons et al., 1998; Páez et al., 2013), social identity approaches to environmental action (Fritsche et al., 2018; Van Zomeren et al., 2010), and studies showing that social support and collective efficacy are crucial in managing climate-related distress and promoting engagement (Fielding and Hornsey, 2016; Mah et al., 2020; Reser and Bradley, 2020). Therefore, the contextual dimension proposed in the present study complements the traditional functional model (problem-, emotion-, and meaning-focused coping) by capturing the social mechanisms through which people regulate emotions and sustain collective engagement in response to the climate emergency.

Nonetheless, it is increasingly recognized that addressing global challenges such as the climate emergency requires not only individual action but also collective, socially coordinated responses (IPCC, 2022; Markowitz and Shariff, 2012). To our knowledge, this social dimension has not been fully integrated into existing coping scales. Nevertheless, social coping strategies—including community engagement, collective advocacy, and peer support—are critical for fostering both individual psychological well-being and broader planetary health (Fielding and Hornsey, 2016). Given this growing awareness, it is essential to explore whether individuals who are emotionally affected by the climate crisis adopt not only personal strategies (e.g., reducing their carbon footprint, changing lifestyle habits), but also socially oriented responses, such as attending demonstrations, joining environmental organizations, or initiating ecological improvements in their workplaces. The extent to which people engage in collective climate action may be influenced by their psychological and emotional responses to the crisis (Cunsolo and Ellis, 2018; Cunsolo et al., 2020).

Emotions and values play a key role in shaping behavior. For instance, climate-related worry tends to be higher among individuals who are more socially engaged and perceive their lives as meaningful—this, in turn, is associated with a stronger sense of personal responsibility and a greater likelihood of taking climate action (Bouman et al., 2020; Ojala et al., 2021; Stanley et al., 2021). Likewise, emotions such as anger and hope have been found to motivate pro-environmental behaviors, including participation in collective action and interest in climate policy (Pihkala, 2022). Collective action also enables individuals to connect with like-minded communities and access social support, which can be particularly beneficial for driving change while safeguarding mental health and well-being (Lawrance et al., 2022). In this sense, Schwartz et al. (2023) study with emerging adults (ages 18–35) showed that being collectively engaged regarding the climate emergency attenuated the positive relationship between climate-change anxiety and general depression symptoms. Other studies observed that young people also emphasized the feeling of being able to do something about the climate emergency when they joined together and protested collectively (Martiskainen et al., 2020; Wallis and Loy, 2021; Watts et al., 2021). Understanding this dual individual-social approach to coping strategies may shed light on the psychological processes underpinning proactive climate-related behaviors and inform interventions to foster collective efficacy in the face of global environmental threats (Mah et al., 2024).

In addition to the functional coping strategies, both for the environment and for the individual, the so-called dysfunctional strategies have already been explored as those that involve reducing or avoiding the perception of the issue rather than addressing it directly (Crandon et al., 2024; Mayer and Smith, 2019; Ojala, 2013). Homburg et al. (2007) elucidated various dysfunctional coping strategies associated with environmental problems, including denial of guilt, relativization, pleasure-seeking, resignation, or wishful thinking. In addition to adaptive or functional coping strategies—those that benefit both the individual and the environment—research has also explored maladaptive or dysfunctional strategies, which involve avoiding or minimizing the perception of the problem rather than addressing it directly (Crandon et al., 2024; Mayer and Smith, 2019; Ojala, 2013). In line with this perspective, the Climate Self-Protection Scale (CSPS) developed by Wullenkord et al. (2021) focuses on defensive psychological mechanisms, such as denial, avoidance, and rationalization, that serve to reduce psychological discomfort but ultimately hinder pro-environmental behavior. While the CSPS provides a valuable framework for understanding barriers to climate action, it does not aim to assess functional coping strategies—neither individual (e.g., information seeking, personal action) nor collective (e.g., social activism, group engagement)—nor does it address the emotional regulation processes that may promote adaptive engagement with the climate crisis.

In the present study, we conceptualize dysfunctional coping not merely as the unfavorable pole of functional strategies, but as a distinct category of disengaged responses with unique motivational and regulatory mechanisms. Previous research has shown that individuals may resort to avoidance, denial, resignation, or hedonistic disengagement to protect themselves from climate-related distress (Clayton, 2020; Homburg et al., 2007; Norgaard, 2011; Ojala, 2013; Wullenkord et al., 2021). Such defensive self-protective strategies can temporarily reduce emotional discomfort but ultimately hinder adaptive adjustment and pro-environmental engagement (Haltinner and Sarathchandra, 2021; Stanley et al., 2021). This theoretical distinction aligns with evidence that maladaptive coping mechanisms constitute qualitatively different psychological processes from adaptive ones, rather than simply representing their inverse (Ojala, 2013; Wullenkord et al., 2021). Accordingly, this conceptualization underlies the third dimension of the CECS, ensuring that the dysfunctional factor represents a theoretically grounded and empirically distinct construct.

In light of theoretical and empirical gaps in the literature, the present research aimed to explore how individuals in Spain cope with the global climate emergency and to develop and validate the Climate Emergency Coping Scale (CECS). Drawing on previous theoretical models (Carver, 1997; Homburg et al., 2007; Ojala, 2012a, 2012b, 2013), we examined how different coping strategies relate to climate emergency perception, pro-environmental engagement, psychological distress (eco-anxiety and eco-worry), and well-being indicators (life satisfaction, resilience, and personal growth).

To evaluate the construct validity of the CECS, we selected criterion variables that have been consistently associated with emotional and behavioral responses to the climate crisis. Specifically, pro-environmental behavior (PEB) was included as a behavioral outcome reflecting adaptive engagement with climate challenges (e.g., Stanley et al., 2021; Wullenkord et al., 2021), and perceived climate action efficacy captured individuals’ sense of agency and perceived capacity to contribute to mitigation efforts (Fritsche et al., 2018; Van Zomeren et al., 2010). Emotional responses and climate change perception were also assessed as key psychological correlates of climate coping (Clayton, 2020; Ojala, 2013).

**Figure 1:**
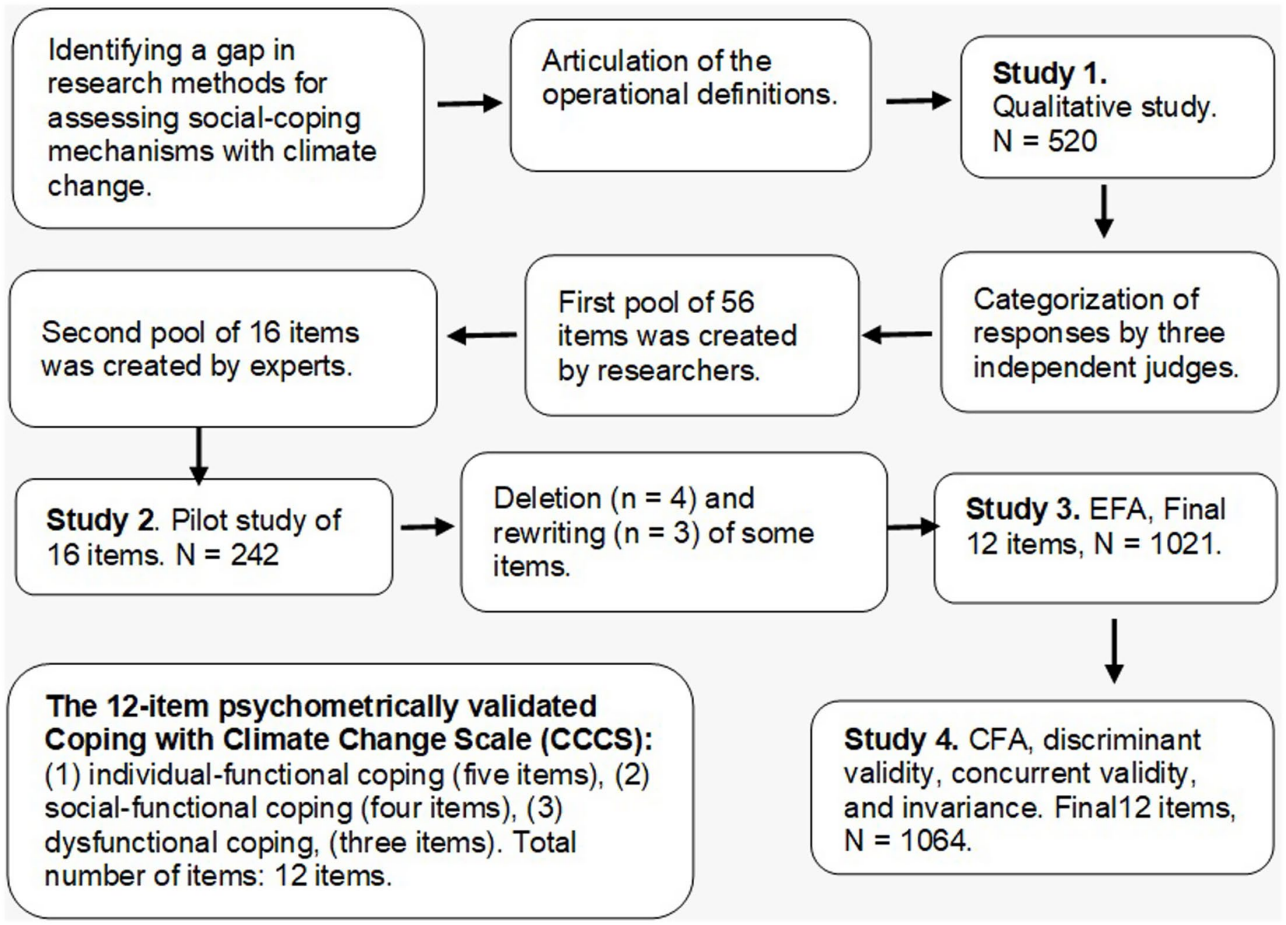
Flowchart presenting the steps of the development and validation of the CECS.

As shown in Figure 1, the research comprised four studies following best practices in scale development (Boateng et al., 2018). Study 1 identified and categorized the main coping strategies related to the climate emergency; Study 2 tested the preliminary psychometric properties of the initial item pool; Study 3 explored the factorial structure of the CECS; and Study 4 confirmed this structure and examined its validity (construct, concurrent, incremental, convergent, and discriminant), as well as measurement invariance across gender, age, socioeconomic status, and education. By developing this scale, we aim to provide a multidimensional tool that captures individual, social, and dysfunctional coping strategies associated with climate-related emotions and engagement.

## 2 Study 1: qualitative study

The aim of Study 1 was to identify and categorize the main coping strategies that individuals use when facing emotions related to the climate emergency. Using qualitative methods, this exploratory study provided the conceptual foundation for item generation in the development of the CECS.

### 2.1 Method

#### 2.1.1 Participants and procedure

Participants were recruited via a combination of social media platforms (e.g., Twitter/X, Instagram, Facebook), environmental websites, and mailing lists of prominent Spanish environmental organizations (e.g., Greenpeace España, Ecologistas en Acción -*Ecologists in action*). The study was advertised through digital flyers and brief explanatory texts, inviting individuals to participate in a survey about their emotional responses and coping mechanisms related to the climate crisis. No monetary or material compensation was offered. Therefore, a snowball sampling method was employed: participants were encouraged to share the survey link with friends or acquaintances, with explicit instructions to include individuals with diverse perspectives on climate issues, ranging from highly concerned to indifferent or even skeptical. This approach resulted in a non-probabilistic convenience sample. Participants accessed the questionnaire online via a Microsoft Forms link and provided informed consent before beginning the survey.

Initially, 520 participants completed the questionnaire. To ensure the relevance of emotional coping mechanisms, we applied an inclusion criterion based on climate-related emotional distress. Specifically, participants were asked an open-ended question: *“Could you describe how you feel about the ecological crisis (climate emergency, biodiversity loss, water scarcity, etc.)?”* Only those who expressed emotional discomfort were included in the final sample. Examples of included responses were “*I feel sad, overwhelmed, and responsible*” or “*I feel helpless and quite useless*.” In contrast, answers such as “*I am not worried. I do not think there is a crisis*” were excluded. This screening resulted in a final sample of 403 participants (35.3% male, 64.3% female), all Spanish residents aged 18 to 81 (M = 42.74, SD = 14.91). Twenty-five percent of participants were members of an environmental organization. Regarding political orientation, 67% identified as left-wing, while 33% identified as center-right (see Table 1).

**Table 1:** Socio-demographic characteristics of samples in Studies 1, 2, 3, and 4.

Socio-demographic characteristics	Study 1 (*n* = 520)	Study 2 (*n* = 242)	Study 3 (*n* = 1,021)	Study 4 (*n* = 1,064)
% Women	52. 1	66.7	51.5	52.3
Mean age (SD)	41.44 (15.492)	36.76 (17.812)	47.51 (15.03)	47.88 (15.016)
Education Level				
% Primary education	2.4	0.8	7.6	7.4
% Secondary education or vocational training	21	27.2	45.9	46.2
% University/College degree and higher	76.6	72	46.5	46.4
Political orientation				
% Left	25.6	24.3	34.8	36.1
% Center Left	26.9	31.7	15.2	14.8
% Center	16.5	27.6	30.3	30.2
% Center Right	9.8	11.5	8.9	8.8
% Right	4	4.9	9.9	9.5
Not answering	17.1		0.9	0.7

### 2.1.2 Measures

**Figure 2:**
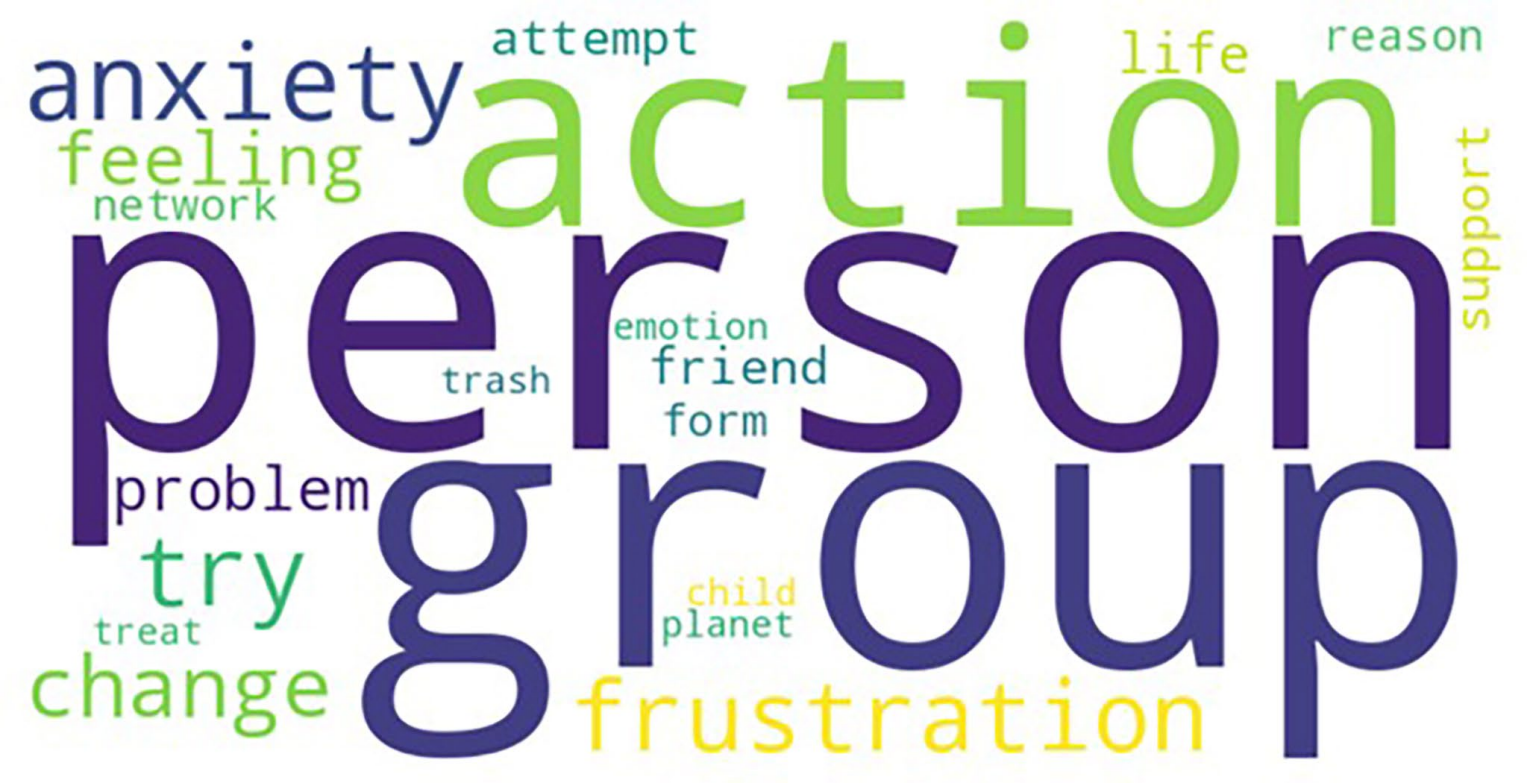
Word cloud representing the most frequently expressed English words (original Spanish text).

A questionnaire was administered in Spanish to begin the development of the scale. Participants were first informed about the study’s objectives and asked for informed consent. Following this, they were presented with a series of sociodemographic questions and one open-ended question. The question was: “*If you have ever felt anxiety, frustration, despair, anger, or similar emotions regarding the environmental crisis, could you tell us what you do to overcome it or what helps you cope with it? What do you rely on (arguments, actions, people, groups, etc.)? Feel free to elaborate as much as you wish.*” Some examples of responses were “Distract me with something else,” “Accept the situation without resignation,” “Act within the scope of my capabilities and my reach,” and “I share my discomfort with people close to me.” Figure 2 illustrates a word cloud representing the most frequently expressed words in the dataset.

### 2.1.3 Data analysis

The subsequent step involved applying a coding system to categorize the data. Given the theoretical review of coping styles in general and those related to climate change (Carver, 1997; Homburg et al., 2007; Ojala, 2012a, 2012b, 2013), a series of codes were described. These keywords or codes served to classify coping strategies in this sample. Atlas.ti 23 software was used to codify this template, which was distributed to three independent experts. They classified the coping strategies of 403 subjects who provided some information following the previously established taxonomy of coping strategies (see Table 2). This deductive process ensured that the data were systematically organized and analyzed, allowing for a comprehensive understanding of the participants’ perspectives and coping mechanisms regarding the ecological crisis. The judges followed a rigorous protocol to ensure consistency and reliability in classifying responses.

**Table 2:** Operational definitions for coding coping mechanisms for climate emotions.

Coping mechanisms	Definition
a. Individual-functional coping	Individual coping mechanisms that benefit the environment and the individual by providing a sense of contributing to the fight against climate change.
a.1. Individual action	Individual actions. i.e., recycling, buying organic or local products, etc.
a.2. Inhibition	Avoid consumption. e.g., reusing, exchanging, or repairing instead of purchasing, eluding private transport, avoiding eating meat. or reducing air conditioning/heating usage.
a.3. Seek for knowledge	Searching for knowledge to lead a more sustainable life. e.g., reading articles or taking courses on sustainable living.
a.4. Connectivity with nature	Seeking contact with nature through hiking, working in a garden, etc., to alleviate the feeling of the climate crisis.
a.5. Acceptance	Cognitive process of recognizing the reality of climate change and taking action to mitigate its effects (instead of resigning).
b. Social-functional coping	Social coping mechanisms that are beneficial to the environment and the individual.
b.1. Collective action	Undertaking collective, community, or social actions like demonstrating or participating in environmental associations.
b.2. Rising awareness	Raising awareness of the climate crisis in others’ contexts, including family, work, or friends.
b.3. Emotional expression	Venting climate emotions with others (crying. shouting).
b.4. Seek for emotional support	Seeking connection with others who share similar concerns about climate change.
c. Dysfunctional coping	Coping mechanisms that are not beneficial to the environment or the individual.
c.1. Avoidance	Avoid thinking about the climate crisis by eluding conversations, news. etc.
c.2. Pleasure	Choosing not to make significant changes in personal comfort levels due to the perception that individual actions will not have a considerable impact.
c.3. Resignation	Sense of hopelessness that significant adverse impacts of climate change are inevitable and that little can be done to prevent them.

Once the process was completed, an expert from the coding team reviewed the templates and, after inter-rater reliability was calculated using α of Krippendorff’s (2019). This reliability coefficient assesses the agreement among inter-rater assessments by means of Atlas.ti 23. The cutoff of Krippendorff’s α ≥ 0.80 was considered good, 0.67 ≤ α < 0.80 was acceptable for tentative conclusions, and α < 0.67 was problematic. This measure assesses the reliability of coding, or how consistently different coders interpret qualitative data. Hence, this measure informs us of the degree of agreement among experts about coping strategies classification.

### 2.2 Results

#### 2.2.1 Coding and reliability

Individual-functional and Social-functional coping had moderate inter-rater reliability (for categories, see Table 2). Specifically, Individual-functional coping was cu-α of Krippendorff = 0.72, indicating an acceptable inter-reliability. Individual-functional coping encompassed the following categories within its semantic domain: individual action, inhibition, seeking knowledge, connectivity with nature, and acceptance. Social-functional coping was cu-α of Krippendorff = 0.68, indicating it was considered an acceptable inter-reliability test by three coders. It included these categories in its semantic domain: collective action, emotional expression, raising awareness, among others, and seeking emotional support. Lastly, the results demonstrated that the inter-rater reliability was deemed suitable for Dysfunctional coping. Dysfunctional coping inter-reliability was a good cu-α of Krippendorff = 0.94. It included the following categories in its semantic domain: avoidance, pleasure, and resignation. Overall, the three semantic domains had a cu-α of Krippendorff = 0.67, which was still acceptable.

#### 2.2.2 Item generation

After ensuring inter-rater reliability (Krippendorff, 2019), a pool of items was created based on the subjects’ responses and the coders’ categorization within the established taxonomy. Responses of subjects in the different categories were analyzed, and the structure of the dimensions was maintained. This structure consisted of a pool of 56 items, created by experts in climate emergency and linguistics (*n* = 3). After eliminating some repetitive items, rewriting others to enhance their comprehension, and ensuring the language was inclusive, a pilot phase (*n* = 242) was conducted to identify potential comprehension issues and estimate the average response time. As a result, some items were rewritten, while others were dropped. A five-point Likert scale was used to measure the degree of agreement with the statements included in the scale, ranging from 1 (never) to 5 (always). The first version of the scale consisted of 16 items, grouped and coded into three preliminary dimensions (see Table 2).

### 2.3 Content validation

To ensure content validity in the initial development of the CECS, as has been observed, we followed a multi-step procedure. First, in Study 1, we collected qualitative data from 520 participants who responded to an open-ended question about how they cope with the emotional impact of the climate crisis. A deductive coding framework was developed based on prior literature on coping strategies, distinguishing between functional-individual, functional-social, and dysfunctional responses. Three trained coders independently categorized the responses, and inter-rater reliability was assessed using Krippendorff’s alpha, with acceptable agreement across categories. Based on these coded responses, a pool of 56 preliminary items was generated.

Then, three external experts—two psychologists with experience in environmental psychology and one linguist—evaluated each item for clarity, relevance, and representativeness of the coping domains. Through this expert judgment process, items were refined, redundant or unclear statements were removed, and a final set of 16 items was selected for pilot testing in Study 2. After analyzing the pilot data, items with poor psychometric properties were eliminated or reworded, resulting in a final 12-item version of the CECS. This process ensured that the scale items were conceptually grounded, clearly worded, and representative of the intended constructs.

## 3 Study 2: pilot study

Study 2 served as a pilot test to examine the preliminary psychometric properties of the initial item pool derived from Study 1. This study aimed to refine the scale by assessing item clarity, redundancy, and internal consistency, and by selecting the most representative items for subsequent analyses.

### 3.1 Method

#### 3.1.1 Participants and procedure

Participants were collected by the snowball sampling method. Participants were recruited using a snowbal sampling method, we had a sample of 242 participants. This sample was a convenience sample. Participants for Study 2 were recruited through online dissemination channels, including university mailing lists, social media platforms (e.g., Twitter, Facebook), and environmental forums in Spain. For the recruitment strategy, no specific inclusion criteria were applied beyond being over 18 years old and providing informed consent. Participation was voluntary and anonymous. No incentives were offered. The participants ranged from 18 to 81 years, with an age of M = 32.74 (SD = 17.81). Regarding income levels, the distribution was as follows: 27.3% of participants reported an income between 1,001 and 1,500 euros per month, 21.5% between 1,501 and 2000 euros per month, and 18.2% between 2001 and 2,500 euros per month. Regarding educational levels, most participants (72.3%) had a university degree, 15.7% had completed secondary education, and 12% had vocational training. As for political orientation, 67% of the participants identified as left-wing, while 33% identified as center-right (see Table 1).

#### 3.1.2 Measures

For the pilot study, the scale’s instructions were drafted as follows: “*Below you will find statements about some of the strategies we can undertake to cope with the emotions generated by the climate crisis, understood as the concept that encompasses climate emergency, desertification, biodiversity loss, abrupt climatic events, and other general environmental problems. Please read each statement carefully and indicate the frequency with which you have approximately performed the following actions in the past year.”* A 1–5 Likert scale of frequency (ranging from ‘never’ to ‘always’) was used for the scale. A total of 16 items were tested.

#### 3.1.3 Data analysis

Items were analyzed by descriptive statistics (mean, standard deviation, skewness, and kurtosis). Subsequently, the item’s structure was analyzed using exploratory factor analysis (EFA) to establish the underlying structure of the items. To ascertain the adequacy of the sampling, the Kaiser-Meyer-Olkin (KMO) and Bartlett’s test of sphericity were employed. These tests are recommended when analyzing a ratio in the context of the analysis being conducted. Following Hair et al. (2010), Bartlett’s test of sphericity must be significant (*p* < 0.05) and the KMO ranges from 0 to 1, but up to 0.5 is an acceptable index (see Williams et al., 2010). Specifically, the unweighted least squares estimation method was used to extract factors and retention of factors with eigenvalues more than 1 (EV > 1). This method is currently the most recommended, as it is particularly effective when working with small samples, even in cases where there are numerous variables and few factors to be retained (Jung, 2013). This method circumvents the occurrence of Heywood cases, which are more prevalent with alternative estimation techniques, as Lloret-Segura et al. (2014) asserted. The Varimax method was used to rotate them, and factor loading was higher than 0.4 when considering an item saturated in a factor. The Varimax method is orthogonal, so it maximizes differences among factors and minimizes variance among items (Lloret-Segura et al., 2014). This orthogonal rotation is recommended when factors are not completely related (Wu, 2014). When an item was saturated in both factors (cross-loaded), it was dropped because there were other items with better loading on both factors (Costello and Osborne, 2005) or were written to improve their wording.

Once factors were estimated, their reliability was calculated by measuring Cronbach’s alpha and the homogeneity index (item-criterion correlation) of the dimensions that emerged (Nunnally, 1978). When the item discriminates adequately, we obtain a positive correlation between the score obtained on the item and between 0.45 and 0.81. Additionally, we considered whether Cronbach’s alpha statistic increased significantly if the item could be removed. The overall reliability of the instrument was also calculated in SPSS 28. Items were analyzed for cross-loading, low loadings, homogeneity index, high asymmetry, high kurtosis, and Cronbach’s alpha if the item is eliminated.

### 3.2 Results

Means, standard deviation, asymmetry, and kurtosis can be observed in Supplementary Table 1. Some items had a higher standard deviation and kurtosis slightly higher than 1. These items could not follow the normal distribution and could be reformulated or deleted. The only item that had higher kurtosis was “*I take actions beneficial to the environment (e.g., recycle, consume responsibly, use public transportation, etc.)*” (M = 3.967, SD = 0.857, As = −0.969, Ku = 1.592) The rest of the items followed the normal distribution since other items had a range of asymmetry and adequate kurtosis. The results showed a KMO value of 0.894, and a Bartlett’s test was significant (*χ*^2^ = 1894.473, df = 120, *p* = 0.001), so the data were adequate for exploratory factor analysis. We performed an exploratory factorial analysis of the items’ factorial structure, and three factors were found. The 16 items explained 61.67% of the variance of the construct. Factor loadings of the items can be observed in Supplementary Table 2.

The first factor explained 41.247% of the construct’s variance, which describes behaviors related to social coping strategies such as support for climate actions and awareness of others to the climate situation, expressing emotions for the climate crisis to alleviate. Nevertheless, there were some items, such as FUN.IND.3 loaded higher than 0.4 in this factor, but showed a higher factor loading in factor 2 FUN.IND5 and FUN.IND.8 was also cross-loading (see Supplementary Table 2), so it is recommended that these items be deleted or reworded for future studies. Additionally, items FUN.IND.6 and FUN.IND.7 were unrelated to the other items since they described individual behavior, although positive ones, so it is recommended to be deleted or reworded. The internal consistency of the scale was α = 0.885. However, observing the factor 1 social coping strategies in Table 3, items FUN.IND.3 and FUN.IND.7 slightly adds internal consistency, which could be deleted to be parsimonious. Hence, the first factor included six items, and after the data analysis, the final number of items in the factors was three.

**Table 3:** Exploratory factor analysis and internal consistency.

	Factor		
	1	2	3
FUN.IND.1.Rw.	**0.631**	0.211	−0.089
FUN.IND.4	**0.765**	0.220	−0.065
FUN.IND.5	**0.757**	0.387	v0.104
FUN.IND.7	**0.604**	0.398	0.044
FUN.IND.8	**0.582**	0.361	−0.021
FUN.SOC.1.Rw.	0.219	**0.686**	−0.014
FUN.SOC.2	0.368	**0.753**	−0.051
FUN.SOC.3	0.344	**0.739**	0.083
FUN.SOC.4	0.463	**0.662**	−0.126
DYSF.1.Rw.	−0.082	−0.032	**0.603**
DYSF.2	0.049	0.013	**0.674**
DYSF.4.Rw.	−0.104	−0.017	**0.760**
Explained variance	42.817%	16.127%	8.586%
Number of items	5	3	4
Alpha’s reliability	0.857	0.712	0.867

Loadings of items in their factor in bold. % of variance explained per factor.

The second factor explained 11.138% of the variance that described individual behaviors such as recycling, saving energy, acceptance, and seeking information for a sustainable life. Nevertheless, DYSF.3 was unrelated to most items’ topics. As can be observed in Table 3, if we deleted this item from the scale, the internal consistency would increase until α = 0.859. Additionally, FUN.IND.1 and FUN.IND.2 had similar wording, so one could be deleted or reworded to be parsimonious. Although the second factor included seven items, the final number of items (after analysis) would be five.

The third factor explained 9.284% of the variance in the behaviors related to avoidance and negation of the climate crisis problem, indicating dysfunctional coping strategies. An example of item DYSF.3, which loaded lower than 0.4 in factor three, exhibited a higher factor loading in factor 2 than in factor 3. It does not make sense to include factor three, which includes functional individual coping strategies, so it is recommended that this item be dropped in the future. This dimension of three items has an internal consistency of α = 0.637. Internal consistency would improve if DYSF.1 were eliminated. It also had a low homogeneity index (r = 0.322). Lastly, item DYSF.2 has a low homogeneity index (r = 0.434). So, this scale did not reach adequate internal consistency (α = 0.637). It is recommended to drop or reword the items and to keep DYSF.4. Therefore, the second factor included three items, and one item was kept DYSF.4 (Supplementary Table 3).

### 3.3 Discussion

Summing up, from the initial list of 16 items, seven were reworded or were excluded due to loading in multiple dimensions (FUN.IND.3, FUN.IND.5, FUN.IND.8, and DYSF.3), unexpected factors (FUN.IND.6, FUN.IND.7), or they did not add internal consistency either homogeneity (FUN.IND.3, FUN.IND.7, DYSF.1, DYSF.2, DYSF.3) which introduced ambiguity regarding the interpretation of the resulting dimensions. The remaining items were included in their hypothesized dimensions. For the following study, new items were created or reworded from the previous one to improve clarity and conciseness.

## 4 Study 3

Building on the results of Study 2, Study 3 aimed to explore the factorial structure of the refined 12-item version of the CECS using exploratory factor analysis. Items showing low item–total correlations or conceptual overlap were removed or adjusted in order to improve the clarity and internal coherence of the scale before confirmatory testing in Study 4.

### 4.1 Method

#### 4.1.1 Participants and procedure

Samples 3 and 4 were collected from a market research company with online panelists. This collaboration enabled us to obtain a representative sample of the Spanish population. After receiving the sample, it was 50% randomly split by the SPSS 28 program. Both samples were compared to test differences in sociodemographic variables. No significant differences were found in gender (*χ*^2^ = 1.206, df = 3, *p* = 0.752), educational level (*χ*^2^ = 0.162, df = 4, *p* = 0.997), political ideology (*χ*^2^ = 2.818; df = 7, *p* = 0.901), activism (*χ*^2^ = 2.325, df = 1, *p* = 0.127).Thus, it could be confirmed that the two subsamples (samples 3 and 4) were similar in their demographic characteristics (see Table 1). The sample size was determined following best practices in scale development (Boateng et al., 2018; Costello and Osborne, 2005). Following the 10:1 participant-to-item rule, at least 120 participants were required for the 12-item scale. Our final samples (Sample 3 = 1,021 for EFA and Sample 4 = 1,064 for CFA and *n* = 1,064 for CFA) largely exceeded this threshold.

Sample 3 (*n* = 1,021) was applied to analyze the final 12 items, the exploratory factor analysis, the improvement of the scale, and the analysis of the factors’ reliability. Sample 3 was composed of 51.2% women, 48.1% men, 3% non-binary, and 4% did not indicate their gender. The age of participants was 47.51 years old, SD = 15.03. Their education level was elementary (7.6%), high school (17.7%), vocational training (28.2%), bachelor’s degree or university degree 29.9%, and Master’s or PhD (16.6%). Regarding political ideology, 5.6% were left extreme, 29.2% were left, 15.2% were center left, 12.9% were center, 8.9% were center right, 8.1.% were right, 1.8% were right extreme, 17.4% do not manifest any ideology, and 9% do not reply to the questions (see Table 1).

#### 4.1.2 Data analysis

Data analysis was replicated from the pilot study (Study 2). Three research team experts created new items and rewrote others of Study 1.

### 4.2 Results

Table 4 shows descriptive results for the proposed pool of 12 items (means, standard deviations, skewness, and kurtosis indexes) in subsample 1 (sample 3). Results showed that all items follow the normal distribution, and the standard deviation of the items is around ±1 of the mean. So, it is not necessary to drop any item from this stage. The results revealed a KMO value of 0.893 and a Bartlett’s test significant (*χ*^2^ = 5625.585, df = 66, *p* = 0.001). Hence, the data were suitable for exploratory factor analysis. The EFA results in subsample 1 (Sample 3) revealed three factors with eigenvalues greater than one, which explained 67.529% of the scale variance.

**Table 4:** Descriptive statistics: means, standard deviations, asymmetry and kurtosis.

Description of items in English and Spanish	M	SD	As	Ku
FUN.IND.1.Rw (En) I take actions that are beneficial to the environment (e.g., recycling. consuming responsibly. using public transportation. etc.) so that I feel I am contributing to alleviating the environmental crisis./*(Sp) Realizo acciones beneficiosas para el medio ambiente (p. ej. reciclo. consumo de forma responsable. uso transporte público etc) para sentir que contribuyo a paliar la crisis ambiental.*	3.69	1.098	−0.760	−0.098
FUN.IND.4 (En) I try not to over-consume water. gasoline. electricity. meat. etc. for environmental reasons./*(Sp) Procuro no consumir en exceso agua. gasolina. luz. carne. etc. por motivos ambientales.*	3.48	1.147	−0.580	−0.322
FUN.IND.5 (En) I inform myself about how to lead a more sustainable life./*(Sp) Me informo sobre cómo llevar una vida más sostenible.*	3.34	1.152	−0.390	−0.517
FUN.IND.7 (En) I seek contact with nature to alleviate my feelings about the climate crisis./*(Sp) Busco el contacto con la naturaleza para aliviar mis sensaciones respecto a la crisis climática.*	3.34	1.191	−0.355	−0.639
FUN.IND.8 (En) I try to accept the climate situation while maintaining a proactive behavior./*(Sp) Trato de aceptar la situación climática manteniendo una conducta proactiva.*	3.26	1.059	−.411	−0.211
FUN.SOC.1.Rw (En) I participate in collective climate actions (e.g., supporting environmental NGOs. attending demonstrations. etc.) to alleviate my distress with climate change./*(Sp) Participo en acciones climáticas colectivas (p. ej. apoyo a ONGs ecologistas. acudo a manifestaciones.* etc.*) para aliviar mi malestar sobre el cambio climático.*	2.20	1.226	0.640	−0.697
FUN.SOC.2 (En) I spend part of my time raising awareness of the climate crisis./*(Sp) Empleo parte de mi tiempo en concienciar a otros sobre la crisis climática.*	2.53	1.196	0.246	−0.865
FUN.SOC.3 (En) I need to express my emotions (anger. sadness. etc.) about the climate crisis to relieve myself./*(Sp) Necesito expresar mis emociones (rabia. tristeza. etc.) sobre la crisis climática para aliviarme.*	2.57	1.198	0.213	−0.865
FUN.SOC.4 (En) I share my uneasiness about the climate crisis with people who feel the same way./*(Sp) Comparto mi malestar sobre la crisis climática con personas que sienten lo mismo.*	3.00	1.261	−0.139	−0.928
DYSF.1.Rw (En) I avoid talking or thinking about the climate crisis./*(Sp) Evito hablar o pensar sobre la crisis climática.*	2.60	1.146	0.289	−0.589
DYSF.2 (En) I think that reducing my level of comfort (using less car. air conditioning. etc.) will not solve environmental problems./*(Sp) Pienso que reducir mi nivel de confort (utilizando menos el coche. el aire acondicionado.* etc.*) no va a solucionar los problemas ambientales.*	2.76	1.189	0.149	−0.799
DYSF.4.Rw (En) I think I can do nothing on an individual level to mitigate a global-scale problem such as the climate crisis./*(Sp) Pienso que no hay nada que yo pueda hacer a nivel individual para mitigar un problema de escala mundial como es la crisis climática.*	2.62	1.180	0.237	−0.683

M, mean; SD, standard deviations; As, asymmetry; and Ku, kurtosis.

Table 3 shows the exploratory factor structure, variance explained by the factors, and Cronbach’s adequate alpha values. All items saturated above 0.3 in factors, there were no mixed-loading items, and no items were loading higher in another factor different from the dimension that item was described (see Table 3).

Tables 3 and 5 show that the first dimension explained 42.817% of the scale variance and included the five items. The factor is called *individual-functional coping. It* refers to actions, behaviors, or practices that individuals adopt to manage their emotional responses and enhance their psychological well-being while actively contributing to climate action. As can be observed in the full scale presented in Appendix A at the end of the article, these items include strategies such as recycling (kept from study 2, but reworded), not consuming many resources (reworded), connectivity, learning about sustainable life, and proactive behavior (that were kept from study 2 and slightly reworded). All items loaded adequately in their factor from 0.582 to 0.765, and their homogeneity index was adequate. Internal consistency was high 0.857, and removing any item to improve it was unnecessary.

**Table 5:** Item analysis of exploratory factor structure in sample 3.

Item	Homogeneity’s index	Cronbach’s Alpha if item deleted	Alpha of dimension
Individual-functional coping strategies			0.857
FUN.IND.1.Rw. (EN) I take	0.606	0.843	
FUN.IND.4	0.712	0.816	
FUN.IND.5	0.774	0.799	
FUN.IND.7	0.644	0.835	
FUN.IND.8	0.626	0.839	
Social-functional coping strategies			0.867
FUN.SOC.1.Rw.	0.654	0.856	
FUN.SOC.2	0.768	0.810	
FUN.SOC.3	0.735	0.823	
FUN.SOC.4	0.717	0.831	
Dysfunctional coping strategies			0.712
DYSF.1.Rw.	0.496	0.678	
DYSF.2	0.533	0.635	
DYSF.4.Rw.	0.586	0.569	

The second dimension explained 16.127% of the scale variance and was composed of 4 items on activities to share information and emotions and raise awareness related to climate emergency. This dimension, called *social-functional coping strategies*, refers to approaches centered on the social self, where individuals address climate emergency’s emotional and psychological challenges through collective and interpersonal actions. These strategies may involve participating in collective efforts, such as attending climate protests or engaging in activities that raise awareness about environmental issues. Additionally, they include seeking emotional support by sharing thoughts and feelings with others with similar concerns about climate emergency. Such strategies emphasize the role of social connection and collective action in fostering resilience and promoting adaptive responses to environmental stressors. All items were loaded adequately from 0.662 to 0.753. The loading was up to 0.50 as varimax rotation in all factors. Internal consistency was adequate, and the homogeneity index of items was correct (see Table 3).

Finally, the third dimension refers to dysfunctional coping strategies, and it explains 8.59% of the scale variance. Dysfunctional coping strategies refer to approaches that are neither beneficial for individuals nor the planet. These strategies often involve maladaptive responses such as succumbing to feelings of resignation and, consequently, taking no action, avoiding the topic of climate emergency altogether, or refusing to alter behaviors if doing so requires a reduction in personal comfort. Such strategies fail to address the underlying challenges of climate emergency and may exacerbate its impacts by fostering inaction and denial. The items describe strategies such as avoidance thinking in the climate crisis, not reducing levels of comfort (e.g., air conditioning), and the thought that nothing can be done to avoid it. All items were loaded adequately from 0.603 to 0.760. Internal consistency was enough, and the homogeneity index of items was correct.

### 4.3 Discussion

In conclusion, the EFA and factors’ reliability analysis generally replicated the three main dimensions identified in the literature review, and interrater agreement and improved reliability and increased explained variance of study 2. This third study showed an adequate % of explained variance, more than 50% for the items, and internal consistency of three dimensions above 0.70 (Nunnally, 1978). Additionally, to assess the validity of this factor structure, a confirmatory factor analysis was conducted, and the relationships between these dimensions with external criteria were examined in subsample 2 (sample 4).

## 5 Study 4

This fourth study aimed to test the factorial structure of the CECS through confirmatory factor analysis and to examine its concurrent and external validity with theoretically related constructs. To this end, several variables were included based on previous research linking coping with environmental and psychological outcomes.

### 5.1 Method

#### 5.1.1 Participants

Sample 4 (*n* = 1,064) was divided into two groups: the other 50% was assigned to Study 3. It was used to perform confirmatory factor analysis, reliability analysis, and validity analysis of the scale. This sample comprised 51.7% of women, 47.7% of men, 2% of non-binary, and 5% who did not reply to this question. The age of the participants was 47.88 years, SD = 15.16. Their education level was elementary (7.4%), high school (18%), vocational training (28.2%), bachelor’s degree or university degree (30.3%), and Master’s or PhD (16.1%). Regarding political ideology, 4.8% were extreme left, 31.3% were left, 14.8% were center left, 14.2% were center, 8.8% were center right, 7.8% were right, 1.7% were extreme right, 16% did not manifest any ideology, and 7% did not reply to the questions.

#### 5.1.2 Measures

##### 5.1.2.1 Pro-environmental behavior (PEB)

To assess the PEB, we adapted 13 items from the Spanish Center for Sociological Research (*Centro de Investigaciones Sociológicas*, CIS). Participants reported the frequency of their engagement in specific behaviors using a 5-point Likert scale ranging from 1 (never) to 5 (always). Example items included: *“I sort glass, cans, plastic, paper, etc., for recycling”* and *“I avoid food waste.”* The scale demonstrated good reliability in the current study (e.g., Cronbach’s alpha, *α* = 0.885).

##### 5.1.2.2 General willingness for environmental behavior scale (GWEBS)

To measure pro-environmental behavioral intention, we used the scale from Vecina et al. (2024), which consists of four items. One example is “*I am willing to voluntarily decrease (consume less).*” Participants rated their agreement with each statement on a 5-point Likert scale ranging from 1 (strongly Disagree) to 5 (strongly Agree). Cronbach’s alpha was α = 0.863.

##### 5.1.2.3 Climate emergency perceptions scale (CEP)

We used the 5-item version from Van Valkengoed et al. (2021) to assess perceptions of the climate emergency. Responses were marked on a 5-point Likert scale from “strongly disagree” to “strongly agree.” An example of an item is ‘*I believe that the climate emergency is real*.’ The Cronbach’s alpha was α = 0.835.

##### 5.1.2.4 The Hogg eco-anxiety scale (HEAS-13)

Eco-anxiety was assessed using the HEAS-13 (Hogg et al., 2021), which consists of 13 items. A 6-month time frame was used in the instructions to ensure the stability of the measure, saying the following: “*Over the last 6 months, how often have you been bothered by the following problems when thinking about climate emergency and other global environmental conditions (*e.g.*, global warming, ecological degradation, resource depletion, species extinction, ozone hole, pollution of the oceans, deforestation)?.”* Participants indicated the frequency with which they experienced each item on a 5-point Likert scale ranging from 0 (never) to 4 (always). The example items are: “*Feeling afraid*” or “*Feeling anxious about the impact of your personal behaviors on the earth.*” The Cronbach’s alpha was α = 0.951.

##### 5.1.2.5 Eco-worry scale

This scale measures the extent of worry and concern individuals have about environmental issues (Parmentier et al., 2024). We used the 5-item version. Responses were marked on a 5-point Likert scale from “strongly disagree” to “strongly agree.” An item example is as follows: “*I worry about the future impact of environmental problems on the planet.”* The Cronbach’s alpha was α = 0.846.

##### 5.1.2.6 Climate agency

To evaluate participants’ beliefs regarding their individual and collective capacity to make a positive impact to mitigate the climate emergency, we used three-item scale measures adapted from Chu and Yang (2020). Participants indicated their responses on a 5-point Likert scale from “strongly disagree” to “strongly agree.” The measure consists of items such as, e.g., “*I believe my actions can have a beneficial influence on climate emergency.*” The Cronbach’s alpha was α = 0.789.

##### 5.1.2.7 The Connor-Davidson resilience scale

This scale measures resilience, defined as the ability to cope with adversity (Connor and Davidson, 2003). We used the short version of four items (from “not true at all” to “true nearly all the time”). One example is” *In general, I look for creative ways to deal with difficult situations.*” The Cronbach’s alpha was α = 0.794.

##### 5.1.2.8 Satisfaction with life scale

This scale measures overall cognitive judgments of one’s life satisfaction (Diener et al., 1985). We used the short version of five items (from “strongly disagree” to “strongly agree”). An example of an item is as follows: “*In most ways, my life is close to my ideal*.” The Cronbach’s alpha was α = 0.861.

##### 5.1.2.9 Personal growth

We used the *Personal Growth subscale* from the *Quiet Ego Scale* (Wayment and Bauer, 2008) to evaluate individuals’ focus on self-improvement and their capacity to integrate personal experiences into a broader meaning and development. Participants responded to the items using a 5-Likert scale format, from 1 (strongly disagree) to 5 (strongly agree). An example is, “*I think it is important to have new experiences that challenge how you think about yourself and the world.*” The Cronbach’s alpha was α = 0.727.

#### 5.1.3 Data analysis

A descriptive analysis of dimensions and variables (mean and standard deviation) was calculated. Additionally, reliability was calculated by Cronbach Alpha, which had a cutoff value of 0.70 (Nunnally, 1978). To test construct validity, a confirmatory factorial analysis was performed in Mplus 8.8 (Muthén and Muthén, 1998-2017). As the nature of the item was ordinal and the sample was vast, the method of estimation chosen was Weighted Least Squared Mean Variance-adjusted (WLSMV). Fit indices used to test model fit were the comparative fit index (CFI), the Tucker-Lewis index (TLI), the root mean squared error of approximation (RMSEA), and the Standardized Root Mean Square Residual (SRMR). Values above 0.90 for CFI and TLI and below 0.08 for RMSEA and SRMR are considered a reasonable model fit. In contrast, stringent recommendations suggest values higher than 0.95 for CFI and TLI and below 0.05 for RMSEA and SRMR (Hu and Bentler, 1999). Evaluation of parameter estimates (e.g., factor loadings) was also considered. We also informed about χ^2^/df, although the limitations of this statistic are known when the sample size is larger due to misspecification of the model (e.g., Hu and Bentler, 1995). The criterion of Fornell and Larcker (1981) has been commonly used to assess the degree of shared variance between the latent variables of the model. According to this criterion, the convergent validity of the measurement model can be assessed by the Average Variance Extracted (AVE) up to 0.5 is acceptable and Composite Reliability (CR) up to 0.7. AVE measures the level of variance captured by a construct versus the level due to measurement error; values above 0.7 are considered very good, whereas a level of 0.5 is acceptable. CR has a less biased reliability estimate than Cronbach’s Alpha, and the acceptable value of CR is 0.7 or above.

Discriminant validity refers to the observation of no association between two constructs that, in theory, should not be related (Tabachnick and Fidell, 2019). The Fornell-Larcker criterion (1981) is one of the most widely used techniques for assessing the discriminant validity of measurement models. It is evidence of discriminant validity that the square root of the AVE values was higher than the correlations of the dimensions of the CECS scale.

To provide evidence of concurrent validity, correlations are examined among dimensions of the scale and the Pro-environmental Behavior Scale (PEB), General Willingness for Environmental Behavior Scale (GWEBS), Climate agency (CA), as well as other variables related to coping strategies for the climate emergency. To test incremental validity, a hierarchical regression analysis was conducted. The eco-worry was included in the first step, Climate Change Perceptions in the second step, and the CECS scale was included in the third step. Outcome variables were: GWEBS and PEB. Considering that Adaptive coping has been associated with pro-environmental behavior (Reser and Bradley, 2020; Ojala, 2013) and with Climate agency—the belief that individual or collective actions can make a difference (Jugert et al., 2016; Swim and Geiger, 2021). Furthermore, functional coping was therefore expected to correlate positively with well-being indicators, whereas dysfunctional coping—marked by avoidance and resignation—was expected to relate negatively to these outcomes and positively to eco-anxiety (Clayton and Karazsia, 2020; Wullenkord et al., 2021).

##### 5.1.3.1 Invariance

This analysis used four structural models, using the lavaan package in R (Rosseel, 2012). In the initial model (Model 0), a single constraint is imposed in which the pattern of factor weights is equal in both groups, known as configural invariance. Next, in Model 1, equality of factor loadings between the groups is imposed, which allows us to assess metric invariance, ensuring that the factor loadings are comparable. Subsequently, in Model 2, equality of intercepts is added to assess scalar invariance, implying that item scores are equivalent across groups without bias. Finally, Model 3 assesses strict invariance by adding equality of item residuals, thus ensuring that residual variances are similar across groups.

Next, measurement invariance is assessed, contrasting whether the items measure the same construct in different groups and whether the differences are not due to item bias (Oppong et al., 2023). The assessment of invariance is fundamental, as it ensures that the construct measured is comparable between groups, allowing valid inferences about the differences observed between them. Using a multiple-group Confirmatory Factor Analysis (CFA), we examined whether the instrument’s factor structure is invariant between the groups compared (gender, age, socioeconomic level, and educational level).

This analysis was performed using four structural models, using the lavaan package in R (Rosseel, 2012). In the initial model (Model 0), a single constraint is imposed in which the pattern of factor weights is equal in both groups, known as configural invariance. Next, in Model 1, equality of factor loadings between the groups is imposed, which allows us to assess metric invariance, ensuring that the factor loadings are comparable. Subsequently, in Model 2, equality of intercepts is added to assess scalar invariance, implying that item scores are equivalent across groups without bias. Finally, Model 3 assesses strict invariance by adding equality of item residuals, thus ensuring that residual variances are similar across groups.

Criteria for assessing compliance with invariance levels are based on the comparison of RMSEA (ΔRMSEA) and CFI (ΔCFI) indices, where differences between models compared with results ΔCFI ≤ 0.010 and ΔRMSEA ≤ 0.015 indicate the absence of significant differences in fit between models (Cheung and Rensvold, 2002). These cutoff points are commonly employed in psychometric studies to ensure consistency in interpreting between-group invariance.

### 5.2 Results

CFA showed evidence of construct validity. Results’ confirmatory factor analysis reproduced the three dimensions and the item distribution derived from the EFA results and results of previous studies (studies 1 and 2). The model showed satisfactory goodness of fit indices (χ^2^ = 432.999, df = 51 *p* < 0.01, χ^2^/df = 8.490; CFI = 0.972; TLI = 0.963; RMSEA = 0.084 90% CI = 0.077–0.091; SRMR = 0.037). Although the statistic χ^2^/df was higher than the cutoff (<5), it was always influenced by the sample size. Hence, the model fits adequately.

Table 6 shows that the factor loadings for the items in the CFA, which ranged from 0.555 to 0.983, were all statistically significant (p < 0.01).

**Table 6:** Confirmatory factor analysis: factor loadings and standard errors in sample 4.

Items	Factor loadings	S.E.
FUN.IND.1.Rw.	0.709	0.018
FUN.IND.4	0.750	0.016
FUN.IND.5	0.886	0.011
FUN.IND.7	0.746	0.016
FUN.IND.8	0.692	0.018
FUN.SOC.1.Rw.	0.767	0.016
FUN.SOC.2	0.862	0.011
FUN.SOC.3	0.785	0.014
FUN.SOC.4	0.847	0.012
DYSF.1.Rw.	0.555	0.027
DYSF.2	0.598	0.027
DYSF.4.Rw.	0.983	0.035

All coefficients are statistically significant (*p* < 0.001).

Table 7 showed descriptive statistics of scale dimensions and related constructs, reliability, and evidence of convergent, discriminant, and concurrent validity. Results showed adequate reliability (see Table 7) for the scale. It was offered α coefficient for all scales (> 0.70), H coefficient (> 0.70), and *ω* (> 0.70) for the scale dimensions. Overall, the dimensions showed adequate reliability. Additionally, the scales that measured related variables also had enough reliability. This table showed the mean, standard deviations, and Cronbach’s alphas for the factors, along with their intercorrelation coefficients. All dimensions of barriers exhibited significant intercorrelations. Nevertheless, dysfunctional coping was negatively related to ‘individual and social coping’, reflecting that people who identify coping in a dysfunctional way were less likely to apply functional coping, both individual and social, and showing that they were opposed. Similarly, functional coping strategies were highly positive and significantly correlated values, whereas correlation values with the dysfunctional factor were lower and significantly negative.

**Table 7:** Descriptive statistics of factors and related variables.

Factors and related variables	Mean	SD	α	H	ω	AVE	Rho	F1	F2	F3
F1-Individual coping	3.414	0.893	0.847	0.89	0.983	**0.58**	**0.871**	**0.76**		
F2-Social coping	2.556	1.008	0.851	0.894	0.995	**0.67**	**0.888**	0.688**	**0.816**	
F3-Dysfunctional coping	2.702	0.924	0.705	0.967	0.981	**0.54**	**0.769**	−0.166**	−0.108**	**0.738**
PEB	3.220	0.739	0.885					0.774**	0.729**	−0.127**
GWEBS	3.760	0.928	0.863					0.659**	0.539**	−0.278**
CCP	4.150	0.829	0.835					0.421**	0.329**	−0.301**
Eco-anxiety	2.350	0.853	0.951					0.314**	0.556**	0.101**
Eco-worry	3.620	0.865	0.846					0.652**	0.657**	−0.214**
CA	3.840	0.943	0.789					0.575**	0.422**	−0.302**
Resilience	3.738	0.771	0.794					0.345**	0.180**	−0.020**
Life satisfaction	3.385	0.860	0.861					0.167**	0.145**	0.000**
Personal Growth	4.056	0.724	0.727					0.340**	0.216**	−0.188**

Evidence of the reliability of CECS factors, and evidence of concurrent and discriminant validity (in bold). **p < 0.01; *p < 0.05 Values in the diagonal are the square root of the AVE value. PEB: Pro-environmental behavior; GWEBS: General Willingness for Environmental Behavior Scale; CCP: Climate Change Perceptions Scale; Climate agency (CA).

To assess the validity of the CECS scale (AERA, APA, NCME, 2014), we correlated the various dimensions of the CECS to other outputs that previous research suggested were related to coping with emotions. Additionally, correlation indexes with CECS scale dimensions, for subsample 2 (sample 4), were all significant, and social and individual coping had higher concurrent validity with the rest of the variables, whereas the opposite, dysfunctional coping, had a significant relationship with all variables but weaker (see Table 7). Specifically, functional coping strategies were positive and significantly related to climate change perceptions, pro-environmental engagement (general willingness to act, pro-environmental behavior, and Climate agency). There were also positive and significant relationships between functional coping well-being indicators (resilience, life satisfaction, and personal growth) and psychological distress indicators (eco-anxiety and eco-worry). Furthermore, there were relationships between dysfunctional coping and climate change perception and pro-environmental engagement, eco-worry, and eco-anxiety. No relationship was found between dysfunctional coping and some well-being indicators, like resilience and life satisfaction, but it was negative and significant with personal growth. The AVE values ranged from 0.666 to 0.544, indicating sufficient convergent validity across all dimensions. Additionally, rho values up to 0.70 of the dimensions were also an indicator of convergent validity (see Table 7). Following Fornell and Larcker’s (1981) criterion is evidence of discriminant validity; the square root of the AVE values (in the diagonal of Table 7) was higher than the correlations of the dimensions of the CECS scale. Additionally, it was used to show discriminant validity from other related constructs, such as resilience, life satisfaction, perspective taking, and personal growth, that showed no relationship or a weak relationship with dimensions of the CECS.

Related to incremental validity, results of hierarchical regression analysis showed that after entering *eco-worry* (step 1), and later CCP (step 2), and, finally, entering CECS (step 3), the results were as follows: concerning GWEBS, variables eco-worry, CCP, and CECS explained 56.5% variance (R^2^ = 0.565, *p* < 0.001). Specifically, CECS’ relationship with GWEBS was positive and significant (β = 0.395, *p* < 0.001), and this variable explained 7.7% of variance (*R^2^* = 7.7, *p* < 0.001). Regarding PEB, the variables explained 62.2% of variance (*R^2^* = 0.622, *p* < 0.001). Specifically, CECS was positively and significantly related to PEB (β = 0.650, *p* < 0.001), explaining 20.9% of the variance (R^2^ = 0.209, *p* < 0.001). So, evidence of incremental validity was shown.

#### 5.2.1 Invariance

The analysis of measurement invariance between gender, age, socioeconomic level, and educational level was based on the estimation of four models, including the configural (Model 0), metric (Model 1), scalar (Model 2), and strict (Model 3), where the results support the notion of measurement invariance across the groups compared. In this sense, the progression in the models, whose fit was significant (see Table 8), and their respective comparison, where the delta values result equal to or less than 0.01 in most of the indices, suggest that the factor structure based on the proposed dimensions was equally valid across the different levels of the analyzed variables. Compliance with measurement invariance allows comparisons and predictions between groups without statistically significant results being affected by biases in the properties of the measurement instrument. This validates the interpretation of the differences observed between groups in the construct evaluated.

**Table 8:** Measurement invariance evaluation by estimating configural (Model 0), metric (model 1), scalar (model 2), and strict (model 3) models across gender, age, socioeconomic status, and educational level variables.

Variable	*χ^2^*	df	CFI	RMSEA	Comparison	ΔCFI	ΔRMSEA
Gender							
Modelo 0	353.23*	102	0.95	0.07			
Modelo 1	364.25*	111	0.95	0.07	M1 vs. M0	<0.01	<0.01
Modelo 2	384.45*	120	0.95	0.07	M2 vs. M1	<0.01	<0.01
Modelo 3	399.79*	232	0.95	0.06	M3 vs. M2	<0.01	<0.01
Age							
Modelo 0	362.47*	102	0.95	0.07			
Modelo 1	370.17*	111	0.95	0.07	M1 vs. M0	<0.01	<0.01
Modelo 2	447.96*	120	0.94	0.07	M2 vs. M1	0.01	<0.01
Modelo 3	457.05*	132	0.94	0.07	M3 vs. M2	<0.01	<0.01
Socioeconomic level							
Modelo 0	474.10*	153	0.94	0.08			
Modelo 1	490.47*	171	0.94	0.07	M1 vs. M0	<0.01	<0.01
Modelo 2	526.26*	189	0.94	0.07	M2 vs. M1	<0.01	<0.01
Modelo 3	574.72*	213	0.94	0.07	M3 vs. M2	<0.01	<0.01
Educational level							
Modelo 0	435.44*	153	0.95	0.07			
Modelo 1	465.97*	171	0.95	0.07	M1 vs. M0	<0.01	<0.01
Modelo 2	536.15*	189	0.94	0.07	M2 vs. M1	<0.01	<0.01
Modelo 3	572.40*	213	0.94	0.07	M3 vs. M2	<0.01	<0.01

M1 vs. M0 represents the comparison between configural and metric invariance. M2 vs. M1 between metric and scalar invariance, and M3 vs. M2 between scalar and strict invariance. *p < 0.05.

Lastly, AVE up to 0.5 and rho up to 0.7 are evidence of convergent validity. Additionally, we conducted a measurement of invariance, which showed no differences among gender, educational level, age, and socioeconomic level.

## 6 General discussion

Global problems require global responses (IPCC, 2023). The climate emergency, as a multidimensional and social threat, demands not only individual change and collective action but also new ways of understanding how individuals and communities psychologically cope with it. Traditional coping scales like Homburg et al. (2007) and Ojala (2012a, 2012b) have often focused on individual cognitive or behavioral responses to stressors; however, in the context of climate change, this is no longer sufficient. It is essential to measure not only individual coping strategies but also social-functional responses, such as engaging with collective movements, seeking group-based support, or participating in community initiatives, as ways to alleviate psychological suffering. Including these dimensions in psychological assessment tools allows researchers and practitioners to evaluate better the full spectrum of emotional responses to climate change—and to identify which coping mechanisms are more effective in buffering the distress associated with ongoing ecological threats. The development and validation of the Climate Emergency Coping Scale (CECS) represent a step forward in this direction, as it incorporates a social coping dimension that reflects the increasing relevance of collective psychological resources in the face of global environmental crises.

In line with this conceptual framework, the findings of the present study provide robust psychometric support for a multidimensional model of coping with climate emergency. Through a series of four studies, the CECS demonstrated a consistent three-factor structure comprising functional-individual, functional-social, and dysfunctional coping. Both exploratory and confirmatory factor analyses confirmed the structural integrity of the scale, with strong and statistically significant factor loadings and adequate model fit. These results underscore the theoretical relevance of distinguishing social coping strategies—those grounded in interpersonal connection, shared identity, and collective efficacy—from strictly individual responses. Moreover, the scale exhibited measurement invariance across gender, age, education, and socioeconomic status, indicating that the CECS performs reliably across diverse demographic subgroups. This reinforces its utility as a culturally appropriate and inclusive tool for assessing emotional responses to climate change in Spanish-speaking populations.

Importantly, the dysfunctional dimension demonstrated psychometric independence from the two functional dimensions. Although it showed moderate negative correlations with functional-individual and functional-social coping, the confirmatory factor analysis supported a three-factor model with good fit indices. This result indicates that dysfunctional coping constitutes a separate latent construct rather than a simple reversal of adaptive strategies. This distinction is consistent with theoretical approaches that conceptualize disengaged or avoidant coping as qualitatively different from active and constructive forms of coping (Ojala, 2013; Wullenkord et al., 2021). Together, these findings reinforce the theoretical and empirical justification for including dysfunctional coping as a distinct dimension of the CECS.

Beyond its structural robustness, the CECS showed strong evidence of external validity, particularly in relation to mental health and climate-related psychological outcomes. Functional coping strategies—both individual and social—were positively associated with pro-environmental behavior, climate change perception, personal growth, resilience, and life satisfaction. These findings suggest that adaptive coping not only promotes engagement with climate action but may also serve as a protective factor for psychological well-being. In contrast, the dysfunctional coping dimension was positively correlated with indicators of distress, such as eco-anxiety and eco-worry, and negatively associated with well-being, aligning with previous studies like Haltinner and Sarathchandra (2021), Ojala (2013, 2018), or Wullenkord et al. (2021). These results showed concurrent validity and underscore the importance of identifying and promoting functional coping strategies—particularly those rooted in social connectedness and collective action—as a way to mitigate the psychological toll of the climate emergency.

In line with this idea, numerous issues commonly framed as “global problems” are, in reality, the cumulative outcome of actions undertaken by individuals, families, communities, private enterprises, and multi-level governmental bodies (Ostrom, 2010). Individuals’ psychological responses to climate emergencies are strongly shaped by the social and communal networks to which they belong, through the availability of collective action pathways and the norms and values upheld by their groups. Emerging evidence suggests that climate-related distress may not only pose a psychological risk, but also act as a catalyst for problem-solving mindsets, increased pro-environmental engagement, and the initiation of collective efforts (Clayton and Karazsia, 2020; Lawrance et al., 2022; Reser et al., 2012; Sciberras and Fernando, 2022; Verplanken and Roy, 2013; Whitmarsh et al., 2022). In this context, social and community networks can modulate how individuals translate climate-related aspirations and anxieties into action—exemplified by the rise of youth-led climate movements such as *Fridays for Future*, which blend emotional expression, peer support, and activism.

Building on this understanding, the CECS introduces an important conceptual advancement by distinguishing functional coping strategies across two domains: individual and social. While previous frameworks—such as those proposed by Ojala (2012a, 2012b) and Homburg et al. (2007)—have offered meaningful typologies of coping (e.g., problem-focused, meaning-focused), they often conflate individual and collective strategies into broad categories. We argue that collective actions (e.g., activism, group engagement, social support) are distinct behaviorally and psychologically from individual strategies (e.g., personal behavior changes, information seeking), and therefore warrant independent measurement. This distinction is particularly salient in the context of the climate emergency, where effective solutions are necessarily systemic and collaborative in nature. Measuring the extent to which individuals engage in social-functional coping allows for a more accurate understanding of how group-based processes support both emotional regulation and civic engagement.

Another relevant instrument that the CECS complements is the Climate Self-Protection Scale (CSPS) by Wullenkord et al. (2021), which offers an important framework for understanding psychological barriers to climate action, but it lacks a focus on functional coping—either individual or collective—and does not address emotional regulation processes that may foster adaptive engagement. In contrast, the CECS explicitly distinguishes between adaptive and maladaptive coping and introduces a social-functional dimension, capturing the relational and collective aspects of how individuals respond psychologically to climate emergency. This conceptual distinction is essential for developing more targeted psychological interventions, educational programs, and public communication strategies that not only aim to reduce distress and defensiveness but also strengthen constructive emotional responses and collective efficacy—both of which are increasingly crucial in facing the ongoing climate crisis.

### 6.1 Practical implications

As climate-related hazards increase in frequency and severity, the emotional consequences of the climate emergency are gaining greater attention in public discourse, education, and psychological research (Hornsey and Fielding, 2020). In this context, the *Climate Emergency Coping Scale* (CECS) can serve as a valuable diagnostic and intervention-oriented tool for clinicians, educators, and policymakers, enabling them to better understand how individuals emotionally respond to climate threats and to guide interventions that promote adaptive coping strategies.

Understanding the coping mechanisms individuals currently use is crucial for evaluating their emotional effectiveness and potential psychological impact. The CECS enables the identification of both functional (individual and social) and dysfunctional coping styles, helping to differentiate between strategies that buffer emotional distress and those that may exacerbate it. In particular, the inclusion of a social-functional dimension makes it possible to assess the role of collective engagement, social support, and group-based action in mitigating climate-related stress. This is especially important for individuals highly engaged in climate activism or working in climate-related fields, as collective coping can reduce the perceived burden of personal responsibility and protect against burnout (Nairn, 2019; Pihkala, 2020).

The clinical relevance of distinguishing between adaptive and maladaptive coping cannot be overstated. Adaptive coping has been associated with psychological resilience, meaning-making, and behavioral engagement, whereas maladaptive strategies—such as avoidance, resignation, and pleasure-seeking—are more likely to maintain emotional arousal, increase stress, and undermine climate engagement (Bell et al., 2001; Gross and John, 2003). Addressing these patterns through early detection and targeted intervention is essential for reducing climate anxiety, particularly in vulnerable populations such as adolescents and young adults.

Psychologists play a central role in the development of interventions and communication strategies that foster adaptive responses to climate-related distress (Gifford, 2011; Stern, 2011). The CECS can inform such efforts by helping professionals tailor their strategies to individuals’ predominant coping styles. For example, interventions could shift individuals from avoidant to engaged coping, or to strengthen social support structures in communities. Indeed, the presence of a supportive social network has been shown to facilitate help-seeking behaviors and buffer the psychological effects of stress (Filipp et al., 1993).

Finally, it is important to recognize that coping strategies vary in their efficacy, and that individuals’ ability to flexibly adapt their coping approaches is essential for promoting mental health and resilience (Sheppes et al., 2014). The choice of coping strategy is influenced not only by individual traits and stressor intensity (Sheppes et al., 2011), but also by socio-political, economic, and cultural contexts that shape available resources and collective meaning systems (Clayton, 2020). By incorporating these dimensions, the CECS provides a comprehensive framework for both research and practice in the emerging field of climate and mental health.

### 6.2 Limitations and future work

Although the present study provides solid psychometric evidence for the *Climate Emergency Coping Scale* (CECS), several limitations must be acknowledged. First, our sample included only residents of Spain, which may introduce cultural biases and limit the generalizability of the findings. While comparable instruments—such as Ojala’s (2012a, 2012b) scale in Sweden and the recent Italian adaptation by Regnoli et al. (2025)—have been developed in other contexts, the CECS is unique in explicitly distinguishing between individual-functional, social-functional, and dysfunctional coping strategies. This social dimension, in particular, is underrepresented in prior instruments. Therefore, validating the CECS across diverse cultural, socio-political, and economic environments—including both individualistic and collectivist societies—remains essential to assess the cross-cultural relevance and robustness of its multidimensional structure.

Furthermore, we recognize that not all individuals have equal access to social support networks or environmental groups, which may influence their capacity to engage in social-functional coping. Future research should explore how variables such as community infrastructure, political engagement, or socio-economic status mediate the use and effectiveness of social coping strategies. The scale’s applicability should also be tested in groups with diverse perceptions of the climate emergency, including denialist or collapsist populations, as well as among those particularly vulnerable to climate anxiety, such as youth climate activists (Van Valkengoed and Steg, 2019).

A second limitation is the exclusive use of self-report measures, which are inherently subject to biases such as social desirability and response style effects. Likert-type items, while efficient, are subjective and may be interpreted differently across individuals (Daeninck et al., 2023). To enhance ecological validity, future studies should incorporate objective or behavioral indicators, or mixed-methods approaches that combine qualitative and quantitative data. Moreover, our data were cross-sectional, limiting the ability to infer causality. Longitudinal designs would provide a more dynamic picture of how coping strategies evolve over time and in response to specific climate events (e.g., extreme weather or policy changes), and would help establish predictive validity of coping profiles in relation to mental health and pro-environmental behavior (Lazarus, 1998).

Additionally, test–retest reliability should be evaluated in future studies to assess the temporal stability of the CECS (McArdle and Woodcock, 1997). Assessing the consistency of coping strategies over time would offer insight into their long-term psychological impact and responsiveness to intervention. It is also important to examine whether the CECS can detect meaningful change, especially in intervention contexts, such as environmental education or clinical treatment programs. Research should prioritize coping education within environmental and academic settings, particularly among students, who appear to be at greater risk for climate anxiety (Daeninck et al., 2023). As Ojala (2016) and Thew et al. (2021) argue, universities should revise their policies and mental health services to integrate emotional resilience training and climate emergency education across disciplines.

Finally, broader theoretical development is still needed. While the CECS distinguishes between adaptive and maladaptive coping, future work could investigate the motivational and contextual factors influencing strategy selection. Personal traits, perceived efficacy, and access to resources—shaped by socio-political or cultural frameworks (Clayton, 2020)—may all condition coping responses. Identifying these drivers will be key to tailoring interventions that not only reduce distress but also promote constructive emotional engagement and collective efficacy. In sum, the CECS provides a promising foundation for this work, offering a novel and comprehensive tool for capturing how people psychologically navigate the climate emergency.

## 7 Conclusion

This study contributes to expanding the toolkit available for understanding the Psychology of Climate Change, particularly in relation to coping processes. To our knowledge, the Climate Emergency Coping Scale (CECS) is the first instrument to explicitly differentiate social-functional coping strategies from individual-functional and dysfunctional strategies—an important theoretical advancement over prior scales. Across five studies, the CECS demonstrated robust psychometric properties in Spanish-speaking populations. Its three-factor structure was consistently supported through both exploratory and confirmatory factor analyses, with strong factor loadings (ranging from 0.555 to 0.983) and satisfactory model fit. Internal consistency was adequate across all subscales, and validity was supported by both convergent and discriminant indicators, including the Fornell-Larcker criterion. Moreover, the scale showed measurement invariance across gender, age, education, and socioeconomic status, indicating its broad applicability. As such, the CECS offers a reliable and theoretically grounded tool for researchers and practitioners seeking to assess how individuals—and communities—emotionally and behaviorally respond to the climate emergency.

## Data Availability

The datasets presented in this study can be found in online repositories. The names of the repository/repositories and accession number(s) can be found at: https://osf.io/954cb/?view_only=bb8bb1739f5f416e9fac076a0bfd9a03.
