# Supplementary material for: Climate emergency coping scale: development and validation of a multidimensional scale
Source: Front Psychol. 2025 Nov 20;16:1665867. doi: 10.3389/fpsyg.2025.1665867 (PMC12678921; doi:10.3389/fpsyg.2025.1665867)
Supplement: Supplementary file 1 [file Table_1.docx]

**Supplementary material (CECS)**

| **Item** | **M** | **SD** | **As** | **Ku** |
| --- | --- | --- | --- | --- |
| FUN.IND.1- I take actions that are beneficial to the environment (e.g., recycle, consume responsibly, etc.). / *Realizo acciones beneficiosas para el medio ambiente (p. ej., reciclo, consumo de forma responsable, etc)*. | 3.967 | .857 | - .969 | 1.592 |
| FUN.IND.2- I take environmental care into account in the small actions of my daily life. / *Tengo en cuenta el cuidado del medio ambiente en las pequeñas acciones de mi vida cotidiana.* | 3.971 | .860 | - .811 | .841 |
| FUN.SOC.1- I participate in collective climate actions (e.g. support NGOs. attend demonstrations. etc.). / *Participo en acciones climáticas colectivas (p. ej. apoyo a ONGs. acudo a manifestaciones. etc.)* | 2.342 | 1.359 | .548 | -1.057 |
| FUN.SOC.2- I spend part of my time raising awareness of the climate crisis. / *Empleo parte de mi tiempo en concienciar a otros sobre la crisis climática.* | 2.321 | 1.271 | .510 | - .926 |
| FUN.IND.3- I give up comforts so as not to increase my ecological footprint. / *Renuncio a comodidades para no incrementar mi huella ecológica.* | 3.099 | 1.012 | - .176 | - .413 |
| FUN.IND.4- I try not to over-consume water. gasoline. electricity. meat. etc. for environmental reasons. / Procuro no consumir en exceso agua. gasolina. luz. carne. etc. por motivos ambientales. | 3.679 | 1.104 | - .693 | - .153 |
| FUN.IND.5- I inform myself about how to lead a more sustainable life. / *Me informo sobre cómo llevar una vida más sostenible.* | 3.506 | 1.162 | - .437 | - .667 |
| FUN.IND.6- I seek information about the climate crisis and possible solutions. / Busco información sobre la crisis climática y sus posibles soluciones. | 3.177 | 1.229 | - .032 | -1.004 |
| FUN.IND.7- I seek contact with nature to alleviate my feelings about the climate crisis. / *Busco el contacto con la naturaleza para aliviar mis sensaciones respecto a la crisis climática.* | 3.263 | 1.310 | - .253 | -1.008 |
| FUN.SOC.3- I need to express my emotions (anger. sadness. etc.) about the climate crisis to relieve myself. / *Necesito expresar mis emociones (rabia. tristeza. etc.) sobre la crisis climática para aliviarme.* | 2.358 | 1.269 | .573 | - .722 |
| FUN.IND.8- I try to accept the climate situation while maintaining a proactive behavior. / *Trato de aceptar la situación climática manteniendo una conducta proactiva.* | 3.329 | 1.075 | - .325 | - .345 |

**Table sup 1.**

Descriptive statistics: means, standard deviations, asymmetry and kurtosis

| FUN.SOC.4- I share my uneasiness about the climate crisis with people who feel the same way. / Comparto mi malestar sobre la crisis climática con personas que sienten lo mismo. | 3.103 | 1.302 | - .238 | -1.084 |
| --- | --- | --- | --- | --- |
| DYSF.1- I avoid the topic of the climate crisis because it makes my day. / Evito el tema de la crisis climática porque me amarga el día. | 2.218 | 1.191 | .635 | - .599 |
| DYSF.2- I think that reducing my level of comfort (using less car. air conditioning. etc.) will not solve environmental problems. / *Pienso que reducir mi nivel de confort (utilizando menos el coche. el aire acondicionado. etc.) no va a solucionar los problemas ambientales.* | 2.543 | 1.267 | .394 | - .856 |
| DYSF.3-  I consume what I want. even if it aggravates the climate crisis. / *Consumo lo que me apetece. aunque con ello agrave la crisis climática.* | 2.152 | .982 | .799 | .460 |
| DYSF.4- I think there is nothing I can do to solve the climate crisis. / *Pienso que no hay nada que yo pueda hacer para solucionar la crisis climática.* | 2.370 | 1.169 | .575 | - .449 |

Note. M=mean, SD=standard deviations, As=asymmetry and Ku=kurtosis

**Table 2 Sup.**

Exploratory Factor Analysis, % variance explained per factor, and internal consistency

|  | | Factor | |
| --- | --- | --- | --- |
|  | 1 | 2 | 3 |
| FUN.IND.1 | .137 | **.713** | - .086 |
| FUN.IND.2 | .168 | **.754** | - .031 |
| FUN.SOC.1 | **.762** | .185 | - .088 |
| FUN.SOC.2 | **.760** | .190 | - .155 |
| FUN.IND.3 | **.468** | **.537** | - .111 |
| FUN.IND.4 | .314 | **.589** | - .082 |
| FUN.IND.5 | **.522** | **.601** | - .182 |
| FUN.IND.6 | **.702** | .339 | - .205 |
| FUN.IND.7 | **.576** | .291 | - .059 |
| FUN.SOC.3 | **.788** | .102 | .025 |
| FUN.IND.8 | **.477** | **.498** | - .198 |
| FUN.SOC.4 | **.664** | .283 | - .048 |
| DYSF.1 | .034 | - .069 | **.442** |
| DYSF.2 | - .138 | - .071 | **.569** |
| DYSF.3 | - .200 | **- .497** | **.398** |
| DYSF.4 | - .137 | - .080 | **.853** |
| Number of items | 6 | 7 | 3 |
| Alpha the Cronbach per dimension | .885 | .694 | .637 |
| % variance explained | 41.247 | 11.138 | 9.284 |

| **Table 3 Sup.**  Item analysis of exploratory factor structure: homegeneity index and Cronbach alpha’s if item is deleted. | | | |
| --- | --- | --- | --- |
| **Item** | **Homogeneity's index** | **Cronbach’s alpha if item deleted** | **Dimension’s alpha** |
| **Factor 1. Social-Functional coping strategies** |  |  | .888 |
| FUN.SOC.1 | .738 | .865 |  |
| FUN.SOC.2 | .744 | .864 |  |
| FUN.IND.3 | **.566** | **.885** |  |
| FUN.IND.6 | .734 | .866 |  |
| FUN.IND.7 | **.600** | **.882** |  |
| FUN.SOC.3 | .717 | .867 |  |
| FUN.SOC.4 | .678 | .872 |  |
| **Factor 2. Dysfunctional coping strategies** |  |  | .637 |
| DYSF.1 | **.322** | **.699** |  |
| DYSF.2 | **.434** | **.558** |  |
| DYSF.4 | .604 | .314 |  |
| **Factor 3. Individual-Functional coping strategies** |  |  | .694 |
| FUN.IND.1 | .583 | .621 |  |
| FUN.IND.2 | .597 | .617 |  |
| FUN.IND.3 | .640 | .594 |  |
| FUN.IND.4 | .601 | .600 |  |
| FUN.IND.5 | .684 | .569 |  |
| FUN.IND.8 | .588 | .606 |  |
| DYSF.3 | **- .554** | **.859** |  |

Note. Items that could be removed in bold.
