# Supplementary material for: Climate emergency coping scale: development and validation of a multidimensional scale
Source: Front Psychol. 2025 Nov 20;16:1665867. doi: 10.3389/fpsyg.2025.1665867 (PMC12678921; doi:10.3389/fpsyg.2025.1665867)
Supplement: Supplementary file 2 [file Supplementary_file_1.pdf]

Appendix A

Climate Emergency Coping Scale (CECS)

C. Díaz-Silveira, F. Latorre, P. Ganitsky-Chemby, F. Burgos-Julián, M.L. Vecina

(En) Below are some common ways people cope with the emotional distress associated with thinking about the climate crisis. These may involve thoughts, emotions, or actions aimed at alleviating climate-related discomfort. Please indicate how often you tend to use each of the following strategies when experiencing such emotions.

(Sp) A continuación se presentan algunas formas habituales en las que las personas afrontan el malestar emocional que les provoca pensar en la crisis climática. Estas formas pueden incluir pensamientos, emociones o acciones dirigidas a aliviar el malestar relacionado con el clima. Por favor, indica con qué frecuencia sueles recurrir a cada una de las siguientes estrategias cuando piensas sobre la crisis climática:

1 = Never 2 = Almost never 3 = Sometimes 4 = Almost always 5 = Always

1 = Nunca 2 = Casi nunca 3 = Algunas veces 4 = Casi siempre 5 = Siempre

1. (En) I take actions that are beneficial to the environment (e.g., recycling, consuming responsibly, using public transportation, etc.) so that I feel I am contributing to alleviating the environmental crisis./ *(Sp) Realizo acciones beneficiosas para el medio ambiente (p. ej., reciclo, consumo de forma responsable, uso transporte público etc) para sentir que contribuyo a paliar la crisis ambiental.*
2. (En) I try not to over-consume water, gasoline, electricity, meat, etc. for environmental reasons. / *(Sp) Procuro no consumir en exceso agua, gasolina, luz, carne, etc. por motivos ambientales.*

3. (En) I inform myself about how to lead a more sustainable life. / *(Sp) Me informo sobre cómo llevar una vida más sostenible.*

4. (En) I seek contact with nature to alleviate my feelings about the climate crisis. / *(Sp) Busco el contacto con la naturaleza para aliviar mis sensaciones respecto a la crisis climática.*

5. (En) I try to accept the climate situation while maintaining a proactive behavior. / *(Sp) Trato de aceptar la situación climática manteniendo una conducta proactiva.*

6. (En) I participate in collective climate actions (e.g., supporting environmental NGOs, attending demonstrations, etc.) to alleviate my distress with climate emergency. / *(Sp) Participo en acciones climáticas colectivas (p. ej. apoyo a ONGs ecologistas, acudo a manifestaciones, etc.) para aliviar mi malestar sobre el cambio climático.*

7. (En) I spend part of my time raising awareness of the climate crisis. / *(Sp) Empleo parte de mi tiempo en concienciar a otros sobre la crisis climática.*

8. (En) I need to express my emotions (anger, sadness, etc.) about the climate crisis to relieve myself. / *(Sp) Necesito expresar mis emociones (rabia, tristeza, etc.) sobre la crisis climática para aliviarme.*

9. (En) I share my uneasiness about the climate crisis with people who feel the same way. / *(Sp) Comparto mi malestar sobre la crisis climática con personas que sienten lo mismo.*

10. (En) I avoid talking or thinking about the climate crisis. / *(Sp) Evito hablar o pensar sobre la crisis climática.*

11. (En) I think that reducing my level of comfort (using less car, air conditioning, etc.) will not solve environmental problems. / *(Sp) Pienso que reducir mi nivel de confort (utilizando menos el coche, el aire acondicionado, etc.) no va a solucionar los problemas ambientales.*

12. (En) I think I can do nothing on an individual level to mitigate a global-scale problem such as the climate crisis./ *(Sp) Pienso que no hay nada que yo pueda hacer a nivel individual para mitigar un problema de escala mundial como es la crisis climática.*
